# Patient Safety in Family Medicine through the Eyes of People with Chronic Conditions in Slovenia

**DOI:** 10.2478/sjph-2026-0012

**Published:** 2026-06-01

**Authors:** Staša Grabar, Zalika Klemenc-Ketiš

**Affiliations:** University of Maribor, Faculty of Medicine, Department of Family Medicine, Taborska ulica 8, 2000 Maribor, Slovenia; Community Health Centre Murska Sobota, Grajska ulica 24, 9000 Murska Sobota, Slovenia; University of Ljubljana, Faculty of Medicine, Department of Family Medicine, Poljanski nasip 58, 1000 Ljubljana, Slovenia; Community Health Centre Ljubljana, Metelkova ulica 9, 1000 Ljubljana, Slovenia

**Keywords:** Patient safety, Family medicine, Primary care, Polypharmacy, Communication, PaRIS, Slovenia, varnost bolnikov, družinska medicina, primarno zdravstveno varstvo, polifarmacija, komunikacija, PaRIS, Slovenija

## Abstract

**Introduction:**

Patient safety is a key dimension of healthcare quality, although most research has focused on hospital settings. This study analysed patient-reported experiences related to safety in Slovenian family medicine using data from the OECD PaRIS survey.

**Methods:**

A cross-sectional study was conducted in 2023 among 70 family medicine practices in Slovenia. Data were collected using two validated questionnaires: the PaRIS Primary Care Patient Questionnaire (PaRIS-PQ) and the Primary Care Practice Questionnaire (PaRIS-PCPQ). Items Q69–Q75 of the PaRIS-PQ were analysed to assess experiences related to the use of medicines, medication review, repetition of information, adverse events, encouragement to express concerns, and barriers to care. Descriptive statistics, chi-square tests, correlation analysis, and binary logistic regression were used in data analysis.

**Results:**

Respondents with chronic conditions totalled 2,133 (mean age 63.1 ± 10.9 years; 62.7% female). Polypharmacy (≥ 3 medications) occurred in 27% of respondents; only 19.7% had received a medication review in the previous 12 months. Seventeen percent reported experiencing at least one adverse event. Significant predictors of reported adverse events included repeated requests for personal medical information (p < 0.001), lack of encouragement to express concerns (p = 0.008), and transport barriers to accessing care (p = 0.024). The regression model explained a small proportion of the variance (Nagelkerke R^2^ = 0.02).

**Conclusions:**

Patient safety in Slovenian family medicine shows both strengths and areas requiring improvement. Communication processes, access barriers, and coordination of information appear to play an important role in perceived safety. Strengthening medication review practices and improving digital interoperability across healthcare settings may contribute to safer care in family medicine. Patient-reported measures such as PaRIS provide valuable insights for monitoring and improving patient safety in family medicine.

## Izvleček

**Uvod:**

Varnost bolnikov je ključna razsežnost kakovosti zdravstvene oskrbe, vendar se je večina dosedanjih raziskav osredotočala predvsem na bolnišnično okolje. Ta raziskava analizira izkušnje bolnikov, povezane z varnostjo v slovenski družinski medicini, na podlagi podatkov raziskave OECD PaRIS.

**Metode:**

Leta 2023 je bila izvedena presečna raziskava v 70 ambulantah družinske medicine v Sloveniji. Podatki so bili zbrani z dvema validiranima vprašalnikoma: PaRIS vprašalnikom za bolnike v primarnem zdravstvu (PaRIS-PQ) in vprašalnikom za ambulante primarnega zdravstva (PaRIS-PCPQ). Za analizo so bila uporabljena vprašanja Q69–Q75 iz vprašalnika PaRIS-PQ, ki obravnavajo uporabo zdravil, pregled terapije, ponavljanje zdravstvenih informacij, neželene dogodke, spodbujanje bolnikov k izražanju skrbi ter ovire pri dostopu do zdravstvene oskrbe. Podatki so bili analizirani z deskriptivno statistiko, hi-kvadrat testom, korelacijsko analizo in binarno logistično regresijo.

**Rezultati:**

V raziskavo je bilo vključenih 2133 pacientov s kroničnimi boleznimi (povprečna starost 63,1 ± 10,9 let; 62,7 % žensk). Polifarmacija (≥ 3 zdravila) je bila prisotna pri 27 % anketirancev, medtem ko je le 19,7 % bolnikov poročalo o opravljenem pregledu terapije v zadnjih 12 mesecih. Sedemnajst odstotkov bolnikov je poročalo o vsaj enem neželenem dogodku. Pomembni napovedniki poročanih neželenih dogodkov so bili ponavljajoče zahteve po posredovanju osebnih zdravstvenih informacij (p < 0,001), pomanjkanje spodbujanja bolnikov k izražanju skrbi (p = 0,008) ter transportne ovire pri dostopu do zdravstvene oskrbe (p = 0,024). Regresijski model je pojasnil majhen delež variance (Nagelkerke R^2^ = 0,02).

**Zaključki:**

Varnost bolnikov v slovenski družinski medicini kaže tako prednosti kot tudi področja, ki zahtevajo izboljšave. Komunikacijski procesi, ovire pri dostopu do zdravstvene oskrbe ter usklajevanje informacij imajo pomembno vlogo pri zaznani varnosti. Krepitev sistematičnega pregleda terapije in izboljšanje digitalne interoperabilnosti med zdravstvenimi sistemi bi lahko prispevala k večji varnosti v primarnem zdravstvu. Bolnikom poročani kazalniki, kot je raziskava PaRIS, ponujajo pomemben vpogled v spremljanje in izboljševanje kakovosti in varnosti v družinski medicini.

## 1 INTRODUCTION

Patient safety is widely recognised as a core component of healthcare quality and an essential dimension of effective health systems. Although patient-safety research has historically focused on hospitals, evidence shows that primary care also faces important safety challenges, including delayed diagnoses, communication failures, incomplete medication reviews, and barriers to access. Despite its central role in health systems, patient safety in primary care remains less extensively studied than safety in hospital settings (1, 2).

According to the World Health Organization (WHO), about 10% of patients globally experience healthcare errors (3). In primary care, risks arise from missed diagnoses, prescribing errors, communication failures, and limited access to services (3,4,5,6). The WHO’s Safer Primary Care manual highlights that safety in these settings requires a distinct approach from hospitals (3).

Diagnostic errors are leading hazards in family medicine (6, 7). Prescribing errors also pose substantial risks; although digital tools reduce serious medication mistakes by over 50%, prescribing remains a key source of harm (8, 9). Preventable drug-related adverse events occur in about 3% of patients, with one-quarter causing serious harm (10). Globally, medical errors lead to millions of preventable injuries each year (11,12,13). The landmark To Err Is Human report estimated 44,000–98,000 US deaths annually from medical errors (14), sparking the modern safety movement (15, 16). In primary care, safety is hindered by fragmented information systems and rising workloads (17,18,19). Physician burnout increases the risk of errors (20), while vulnerable populations—particularly older adults and people with chronic conditions—face higher exposure (21, 22).

In Slovenia, research shows a positive association between safety climate and outcomes, although regional and institutional variation persists (23, 24). Tools such as the Safety Attitudes Questionnaire (SAQ-SF) (25) and the Medical Office Survey on Patient Safety Culture (MOSPSC) have been used. A recent study reported an overall safety culture score of 59.6%, with strong domains in information exchange (93.5%) and organisational learning (90.2%), but weaknesses in work pressure (10.7%) and leadership support (27.1%) (26).

In recent years, patient-reported outcome measures (PROMs) and patient-reported experience measures (PREMs) have gained importance as complementary indicators of healthcare quality and safety (27). The OECD Patient-Reported Indicator Surveys (PaRIS) programme provides a unique opportunity to assess the experiences and outcomes of care from the perspective of people with chronic conditions across countries (23, 30,31,32). To our knowledge, patient-reported safety indicators from the PaRIS survey have not yet been analysed in Slovenia using this approach.

The aim of this study was therefore to examine patient-reported safety indicators in Slovenian family medicine among people with chronic conditions, with particular attention to medication burden, medication review, communication and information continuity, and barriers to care. The study also explored predictors of reported adverse events in order to identify areas for improvement in the safety of family medicine practice.

## 2 METHODS

### 2.1 Study design and setting

This cross-sectional study was part of the international Patient-Reported Indicator Surveys (PaRIS) programme, coordinated by the Organization for Economic Co-operation and Development (OECD) (23). The Slovenian part of the study was conducted in 2023 at 70 randomly selected family medicine practices, representing various geographic and demographic areas (28,29,30). The OECD developed the overall PaRIS survey design and conceptual framework in consultation with several international collaborators, including the Netherlands Institute for Health Services Research (NIVEL), Ipsos MORI, the University of Exeter, the Avedis Donabedian Institute, and OptiMedis AG (23).

### 2.2 Participants

#### 2.2.1 Providers

All active family medicine practices in Slovenia (N = 978) were eligible for inclusion according to the national register kept by the Health Insurance Institute of Slovenia. Practices that provided care exclusively to children or to institutionalised patients only were excluded. Participation was voluntary and unincentivised.

#### 2.2.2 Patients

Eligible participants were community-dwelling adults aged 45 years or older with at least one chronic condition and at least one contact with their family physician in the previous 6 months, including face-to-face, telephone, or online contact. People with severe cognitive impairment, terminal illness, or other disabling conditions preventing participation were excluded. For each participating practice with electronic medical records, a random list of 300 eligible patients was generated.

### 2.3 Instruments

Two validated OECD PaRIS instruments were used.

At the practice level, the Primary Care Practice Questionnaire (PaRIS-PCPQ) was administered. The full instrument contains 40 items covering organisational characteristics, workforce composition, care coordination, chronic care management, and the use of electronic health information systems (31). For the purposes of this analysis, the following PCPQ items were used:

(1) practice location,
(2) practice type,
(3) electronic exchange of patient information with external providers,
(4) review of indicators of patient care, and
(5) routine development of patient care plans for groups of patients.

At the patient level, the Primary Care Patient Questionnaire (PaRIS-PQ) was used. The full instrument comprises 121 items addressing sociodemographic characteristics, health behaviours, health status, patient-reported outcomes, and patient experiences of care (32). In the present study, 7 patient-safety-related items from the PaRIS-PQ (Q69–Q75) were analysed, covering:

- number of medications taken regularly,
- medication review in the previous 12 months,
- repetition of medical information,
- reported adverse events,
- whether respondents were encouraged to express concerns,
- foregoing care due to transport difficulties, and
- foregoing care due to medical costs.

Both instruments were translated into Slovenian and culturally adapted using the TRAP-D process (Translation, Review, Adjudication, Pretesting, and Documentation) to ensure linguistic accuracy and conceptual equivalence (29).

### 2.4 Data collection and protection

Data collection took place from April to June 2023. Participation was voluntary and anonymous. The study adhered to the General Data Protection Regulation (EU) 2016/679. All responses were anonymised before analysis, and no personally identifiable data were collected or stored.

#### 2.4.1 Providers

Participating providers received detailed instructions, consent forms, and unique identification codes to maintain confidentiality and to link provider- and patient-level data. Questionnaires were administered electronically or on paper, depending on participant preference.

#### 2.4.2 Patients

Participants received clear information about the study’s purpose, a consent form, and a unique identification code to protect their privacy and to link their responses anonymously to their healthcare provider. Participants could complete the questionnaire either electronically or on paper, depending on their preference. Invitations used a multimodal approach: via email, SMS or postal mail. Non-respondents received a total of two reminders within a three-week period.

### 2.5 Variables

The primary outcome variable was reported adverse events derived from PaRIS-PQ item Q72. This item asked respondents how often they had experienced harmful or adverse events related to their care. For regression analysis, this variable was dichotomised into the presence of adverse events versus no adverse events, as specified below.

Independent variables at the patient level included:

- number of regularly taken medications,
- medication review within the previous 12 months,
- repetition of medical information to healthcare providers,
- lack of encouragement for respondents to express their concerns,
- barriers to care due to transport difficulties, and
- barriers to care due to medical costs.
- At the provider level, independent variables included:
- practice location (urban/rural),
- practice type (public/private concession practice), and
- electronic exchange of patient information with external healthcare providers.

### 2.6 Statistical analysis

Descriptive statistics were used to summarise provider-and patient-level characteristics, including frequencies, percentages, means, and standard deviations, as appropriate. Associations between categorical variables were examined using chi-square tests, and associations between ordinal patient-safety indicators were assessed using Spearman’s rank correlation coefficients.

The primary outcome for multivariable analysis was reported adverse events (Q72). For binary logistic regression, responses were dichotomised as follows: respondents reporting adverse events “always”, “often”, or “sometimes” were classified as having experienced an adverse event, whereas those responding “rarely” or “never” were classified as not having experienced an adverse event. Responses marked as “not suitable for me” or missing were excluded from the regression model.

Binary logistic regression was used to identify predictors of reported adverse events. Independent variables included the number of regularly taken medications, medication review within the previous 12 months, repetition of medical information, lack of encouragement for respondents to express concerns, barriers to care due to transport difficulties, and barriers to care due to medical costs. Results are presented as regression coefficients (B), standard errors (SE), Wald chi-square statistics, p-values, odds ratios (ORs), and 95% confidence intervals (CIs).

Because respondents were clustered within practices, a multilevel mixed-effects logistic regression model with a random intercept for practice was applied in accordance with OECD PaRIS analytic procedures. Model fit was assessed using the model chi-square statistic and Nagelkerke R^2^. Statistical significance was set at p < 0.05. Analyses were performed in SPSS version 31.

### 2.7 Ethical considerations

The study was conducted in accordance with the principles of the Declaration of Helsinki and approved by the National Medical Ethics Committee of the Republic of Slovenia (No. 0120-260/2021/3). All participants provided informed consent before inclusion in the study.

## 3 RESULTS

### 3.1 Demographic characteristics of the participants

#### 3.1.1 Providers

Seventy family medicine practices with at least one registered person with a chronic condition took part in the study. Table 1 presents the distribution of provider-level characteristics among participating family medicine practices.

**Table 1.** Characteristics of providers.

**Variables**	**Description**	**N (number of practices)**	**%**
**Practice location**	Urban	49	70.0
	Rural	21	30.0
**Type of practice**	Public	60	85.7
	Private	10	14.3
**Electronic exchange of patient information**	Yes	38	54.3
	No	32	45.7
**Review of indicators of patient care**	Yes	44	62.9
	No	26	37.1
**Routine development of patient care plans for groups of patients**	Yes	37	53.6
	No	33	46.4

#### 3.1.2 Patients

Overall, 2,133 respondents with at least one chronic condition returned the questionnaire, representing 64.6% of all respondents in the Slovenian PaRIS sample. Table 2 presents the distribution of patient-level demographic, socioeconomic, and lifestyle characteristics among respondents with chronic conditions who participated in the study.

**Table 2.** Characteristics of patients.

**Variables**	**N (number of practices)**	**%**
**Sex**		
Female	1268	62.7
Male	754	37.3
**Age (years) categories**		
45–54	505	23.7
55–74	1355	63.6
75 +	271	12.7
**Higher education**		
Yes	1247	61.7
No	774	38.3
**Employment**		
Yes	886	43.9
No	1133	56.1
**Income**		
Low	758	43.1
Medium	597	34.0
High	403	22.9
**Living place**		
Urban	1094	54.2
Rural	923	45.8
**Living with other people**		
Yes	1402	73.1
No	515	26.9

### 3.2 Patient safety indicators

Table 3 summarises the responses from respondents regarding patient safety indicators, including the pattern of medication use, medication review by healthcare professionals, communication safety, adverse events, encouragement of respondents to express concerns, and access barriers such as transport and cost.

**Table 3.** Distribution of responses to patient safety indicators.

**Indicator**	**Category**	**N (number of practices)**	**%**
**Number of medications regularly taken**	Does not take any medicine	282	8.5
	1-2	890	27.0
	3-4	602	18.2
	5-9	257	7.8
	10 or more	33	1.0
**Medication review in the last 12 months**	Yes	650	19.7
	No	991	30.0
	I’m not sure	135	4.1
**Repetition of medical information**	Yes, definitely	155	4.7
	Yes, to some extent	246	7.4
	Neither	849	25.7
	Not at all	1439	43.6
	I’m not sure	295	8.9
**Frequency of adverse events**	Always	22	0.7
	Often	157	4.8
	Sometimes	380	11.5
	Rarely	721	21.8
	Never	1596	48.3
	Not applicable	101	3.1
**Encouragement to express concerns**	Always	7	0.2
	Often	95	2.9
	Sometimes	299	9.1
	Rarely	457	13.8
	Never	1967	59.6
	Not applicable	147	4.5
**Foregoing care due to transport difficulties**	Always	3	0.1
	Often	33	1.0
	Sometimes	103	3.1
	Rarely	230	7.0
	Never	1695	75.9
	Not applicable	96	2.9
**Foregoing care due to medical costs**	Always	91	2.8
	Often	123	3.7
	Sometimes	313	9.5
	Rarely	400	12.1
	Never	1700	51.5
	Not applicable	337	10.1

**Table 4.** Cross-tabulation of polypharmacy and medication review, NA – not applicable.

**Number of medications**	**Medication review: Yes (N, %)**	**Medication review: No (N, %)**	**Not sure (N, %)**	**Total (N)**
**No medication**	NA	NA	NA	282
**1-2**	277 (31.2%)	554 (62.2%)	56 (6.6%)	890
**3-4**	240 (39.9%)	306 (50.8%)	54 (9.0%)	602
**5-9**	119 (46.3%)	120 (46.7%)	18 (7.0%)	257
**≥ 10**	14 (43.8%)	11 (34.4%)	7 (21.9%)	33
**Total**	650 (36.6%)	991 (55.8%)	135 (7.6%)	1776

Cross-tabulation analyses showed statistically significant associations between medication burden and several patient safety indicators. Respondents taking a higher number of medications were more likely to report having had a medication review, to report repeating their medical information, and to report adverse events. They were also more likely to report barriers to care related to transport and costs (all p < 0.001). These findings suggest that respondents with a greater medication burden may also face greater safety-related vulnerability.

Spearman’s correlation analyses showed that repetition of medical information was positively associated with reported adverse events (r_s = 0.16, p < 0.001). Transport barriers (r_s = 0.12, p < 0.001) and cost barriers (r_s = 0.15, p < 0.001) were also positively associated with adverse events. Encouragement to express concerns was negatively associated with adverse events (r_s = −0.08, p = 0.004). Although these associations were statistically significant, their magnitude was weak and should therefore be interpreted with caution (Table 5).

**Table 5.** Spearman’s rank correlations between patient safety indicators.

**Variable**	**Repetition**	**Adverse events**	**Encouragement**	**Transport barriers**	**Cost barriers**
**Repetition**	1.00	0.16	-0.05	0.09	0.11
**Adverse events**	0.16	1.00	-0.08	0.12	0.15
**Encouragement**	-0.05	-0.08	1.0	-0.04	-0.03
**Transport barriers**	0.09	0.12	-0.04	1.00	0.22
**Cost barriers**	0.11	0.15	-0.03	0.22	1.00

A multivariable logistic regression analysis was conducted to identify predictors of reported adverse events. The overall regression model was statistically significant (χ^2^(6) = 24.8, p < 0.001), although the proportion of explained variance was low (Nagelkerke R^2^ = 0.02) (Table 6).

**Table 6.** Logistic regression analysis of predictors of adverse events.

**Predictor**	**B (SE)**	**Wald χ^2^**	**p-value**	**OR**	**95% CI**
**Number of medications / Polypharmacy**	-0.28 (0.34)	0.69	0.492	0.75	0.34-1.62
**Medication review**	-0.27 (0.30)	0.83	0.406	0.76	0.42-1.36
**Repetition of information**	0.42 (0.17)	6.13	< 0.001	1.53	1.20-1.95
**Encouragement to express concerns**	0.76 (0.47)	2.65	0.008	2.14	1.14-4.03
**Transport barriers**	0.32 (0.21)	2.26	0.024	1.1	1.02-1.95
**Cost barriers**	-0.07 (0.05)	1.66	0.097	0.93	0.86-1.99

Legend: SE– standard error; OR – odds ratio; C.I – confidence interval

Among the examined predictors, repetition of medical information was significantly associated with a higher likelihood of reporting adverse events (B = 0.42, SE = 0.17, Wald χ^2^ = 6.13, p < 0.001). Respondents who reported frequently having to repeat their medical information had approximately 1.53 times higher odds of reporting adverse events compared with those who did not (OR = 1.53, 95% CI 1.20–1.95). Similarly, lack of encouragement to express concerns was associated with increased odds of adverse events (B = 0.76, SE = 0.47, Wald χ^2^ = 2.65, p = 0.008). Respondents who reported less encouragement to express concerns had 2.14 times higher odds of reporting adverse events (OR = 2.14, 95% CI 1.14–4.03). Transport barriers to care were also a significant predictor (B = 0.32, SE = 0.21, Wald χ^2^ = 2.26, p = 0.024), indicating that respondents facing transportation difficulties had slightly higher odds of reporting adverse events (OR = 1.10, 95% CI 1.02–1.95). In contrast, the number of medications, medication review within the previous 12 months, and cost-related barriers to care were not statistically significant predictors in the adjusted model (all p > 0.05). For example, the number of medications taken regularly showed no significant association with adverse events (B = −0.28, SE = 0.34, p = 0.492; OR = 0.75, 95% CI 0.34–1.62).

## 4 DISCUSSION

This study examined patient-reported safety indicators in Slovenian family medicine using OECD PaRIS data from people with chronic conditions. The findings point to several relevant safety challenges, particularly in medication-related care, information continuity, patient communication, and access to care. However, the strongest adjusted associations with reported adverse events were observed for repetition of medical information, lack of encouragement for respondents to express their concerns, and transport barriers, whereas medication burden itself was not a significant predictor after adjustment.

### 4.1 Medication safety and polypharmacy

Medication-related safety remains a key concern in primary care, especially among people with chronic conditions and multimorbidity. In our study, a substantial proportion of respondents reported regular medication use, yet only a minority reported having undergone a medication review within the previous 12 months. This finding suggests potential gaps in systematic medication monitoring in family medicine.

Polypharmacy is widely recognised as an important risk factor for adverse drug events, hospitalisations, and inappropriate prescribing, particularly in older populations. Numerous studies have shown that increasing medication burden is associated with a higher risk of medication-related harm (1–2, 4, 10). However, in the present study polypharmacy was not significantly associated with reported adverse events in the adjusted regression model. This suggests that medication burden alone may not fully explain safety outcomes in this dataset. Instead, the relationship between polypharmacy and patient safety may be mediated by other factors, such as the quality of medication review, care coordination, and patient-provider communication.

These findings are consistent with previous research suggesting that medication-related safety risks often arise not only from the number of medications but also from system-level factors such as inadequate medication reconciliation and fragmented care processes (6, 18–19).

### 4.2 Communication and information continuity

One of the strongest predictors of reported adverse events in this study was the need for respondents to repeat medical information that should already be available in the medical record. This finding likely reflects fragmentation of health information systems and limited interoperability between providers. When patient information is not consistently accessible across care settings, the risk of communication errors, duplicated tests, and inappropriate treatment decisions may increase.

Similarly, respondents who reported less encouragement to express their concerns had higher odds of reporting adverse events. This finding highlights the importance of patient engagement and open communication in maintaining safe care processes. Previous studies have shown that when people feel comfortable voicing concerns and participating in decision-making, healthcare teams are more likely to detect potential safety issues early and prevent harm (27, 33–34).

Despite this, a large proportion of respondents reported that they were rarely or never encouraged to express concerns during consultations. This finding suggests that patient empowerment and shared decision-making may still represent an underdeveloped aspect of patient safety culture in primary care.

### 4.3 Access barriers and patient safety

**Transport barriers were also associated with a higher** likelihood of reporting adverse events. Although Slovenia has a largely publicly funded healthcare system, physical access to care may still represent an important determinant of patient safety, particularly for older adults and people with multimorbidity.

Barriers to transport may lead to delayed consultations, missed follow-up appointments, and reduced continuity of care, all of which may increase the risk of adverse outcomes. By contrast, cost-related barriers were not statistically significant predictors in the adjusted model, suggesting that in the Slovenian context physical accessibility may play a more important role than financial barriers in shaping patient safety experiences.

### 4.4 Interpretation of patient-reported adverse events

Approximately one in six respondents reported experiencing an adverse event at least occasionally. These results should be interpreted cautiously, as the survey item captures patient-reported experiences rather than clinically validated safety incidents. Patient-reported safety indicators provide valuable insight into perceived problems in care processes, but they may reflect a broader range of experiences than formally documented medical errors.

Nevertheless, respondent perspectives are increasingly recognised as an important component of safety monitoring, particularly in primary care where many safety events remain undocumented in clinical reporting systems (27, 33–34).

### 4.5 Model performance and interpretation

The regression model explained only a small proportion of the variance in reported adverse events (Nagelkerke R^2^ = 0.02). Although this value appears low, such findings are common in studies examining complex social and behavioural phenomena such as patient safety in primary care. Safety outcomes are influenced by multiple interacting determinants at the patient, provider, organisational, and health-system levels, many of which were not captured in the present analysis.

Similarly, the correlations observed between safety indicators were generally weak. This pattern suggests that patient safety incidents in primary care are multifactorial and cannot be explained by individual indicators alone. Even so, identifying statistically significant predictors can still provide useful insights into areas where improvements in care processes may reduce safety risks.

### 4.6 Strengths and limitations

This study has several strengths. It uses data from the OECD PaRIS programme, applies internationally developed and culturally adapted instruments, and includes a large national sample of Slovenian people with chronic conditions in family medicine.

The study also has important limitations. First, the cross-sectional design does not allow causal inference. Second, all patient-level data were self-reported, meaning that the adverse events captured in this study reflect respondent’ perceptions and experiences rather than clinically validated safety incidents. Third, the dichotomisation of the adverse-event item may have reduced nuance by combining different frequencies of reported events into a binary outcome. Fourth, the observed correlations were weak, and the regression model explained only a small proportion of the variance, indicating limited predictive power. Fifth, participation was voluntary, which raises the possibility of non-response bias, including possible overrepresentation of more engaged practices or respondents with higher health literacy. These factors may limit generalisability.

### 4.7 Implications for practice

Despite these limitations, the findings point to several actionable areas for improvement. First, medication review should be strengthened in routine family medicine care, especially for people with multimorbidity and higher medication burden. Second, digital interoperability and information continuity should be improved to reduce the need for respondents to repeat medical information across providers. Third, communication practices should more consistently support people in expressing concerns, as this may contribute to safer and more person-centred care. Finally, access barriers—particularly transport-related barriers—should be considered within national patient-safety strategies, because safe care depends not only on clinical decisions but also on people’s ability to reach and navigate services.

Regular medication review, better digital information exchange, and incorporation of patient-reported experience and outcome measures into routine quality monitoring may represent feasible system-level steps toward improving patient safety in Slovenian family medicine.

## 5 CONCLUSION

Patient safety in Slovenian family medicine is shaped by multiple interacting factors. In this study, repetition of medical information, limited encouragement for respondents to express their concerns, and transport barriers were associated with higher odds of reported adverse events. Although medication burden was linked to several safety-related indicators in unadjusted analyses, it was not independently associated with adverse events after adjustment. These findings suggest that patient safety in primary care should be understood not only in terms of clinical complexity, but also in terms of communication quality, continuity of information, and access to care.

Strengthening routine medication review, improving digital interoperability, and supporting people’s voice within consultations may help improve safety in family medicine. OECD PaRIS data provide a valuable patient-centred perspective that can inform future quality improvement and national patient-safety policy in Slovenia.
